# Supplementary material for: Graphene Oxide–Platinum Nanoparticle Nanocomposites: A Suitable Biocompatible Therapeutic Agent for Prostate Cancer
Source: Polymers (Basel). 2019 Apr 23;11(4):733. doi: 10.3390/polym11040733 (PMC6523086; doi:10.3390/polym11040733)
Supplement: Supplementary file 1 [file polymers-11-00733-s001.zip › Supplementary-467913/Supplementary caption.docx]

**Supplementary legends**

**Suppl. Figure 1. Characterization of GO and GO-PtNPs by SEM and TEM**

Morphology of GO (A), GO-PtNPs (B), size of GO (C) and GO-PtNPs (D) was analyzed by SEM and TEM respectively. The red circle indicates decoration of PtNPs particles on the surface of graphene sheet (White arrow). The graphene sheet depicted as wrinkled structure. E. The size distribution of GO. F. Size distribution of GO-PtNPs

**Suppl. Figure 2. The cell morphology was analyzed by phase-contrast microscope**

The morphology of LNCaP cells was determined after 24-h exposure to different concentrations of GO (A), GO-PtNPs (B) and PtNPs (C) using light microscope.

**Suppl. Figure 3.**

LNCaP cells was exposed to respective IC50 concentration of GO, GO-PtNPs and PtNPs for 24 h and then ROS was measured using DCFH-DA-FITC by fluorescence microscopy analysis. Scale bar =200 μm.

**Suppl.figure 4**

LNCaP cells was exposed to respective IC50 concentration of GO, GO-PtNPs and PtNPs for 24 h and then MMP was analysed by fluorescence microscopy analysis. Scale bar =200 μm.
