# Supplementary material for: Graphene Oxide–Platinum Nanoparticle Nanocomposites: A Suitable Biocompatible Therapeutic Agent for Prostate Cancer
Source: Polymers (Basel). 2019 Apr 23;11(4):733. doi: 10.3390/polym11040733 (PMC6523086; doi:10.3390/polym11040733)
Supplement: Supplementary file 1 [file polymers-11-00733-s001.zip › Supplementary-467913/Supplementary materials and methods.docx]

2. Materials and Methods

2.1. Materials

Graphite powder, NaOH, KMnO4, NaNO_3_, anhydrous ethanol, 98% H_2_SO_4_, 36% HCl, 30% H_2_O_2_ aqueous solution, and all other chemicals were purchased from Sigma-Aldrich (St. Louis, USA) unless otherwise stated. Penicillin-streptomycin solution, trypsin-EDTA solution, RPMI 1640 medium, and 1% antibiotic-anti-mycotic solution were obtained from Life Technologies/Gibco (Grand Island, NY, USA). Vanillin, fetal bovine serum, and the *in vitro* toxicology assay kit were purchased from Sigma-Aldrich (St. Louis, MO, USA). The CCK-8 cell viability assay kit, lactate dehydrogenase (LDH) cytotoxicity detection kit were purchased from Dojindo (Kumamoto, Japan), ROS assay kit, 2′,7′-dichlorofluorescin diacetate (DCFH2-DA), and Sigma-Aldrich, and Takara Bio (Shiga, Japan), respectively.

2.2. Synthesis and characterization of GO, PtNPs and GO-PtNPs

Graphene sheets were synthesized by Hummers’ method with slight modification as required [8,33]. Graphite (2 g) was mixed with NaNO_3_ (2 g) in H_2_SO_4_ (96 ml) at 0ºC (ice bath), then KMnO_4_ (12 g) was gradually added over 1 h, while maintaining the temperature at 0ºC. After that, the reaction mixture was stirred at 0ºC for 2 h, followed by at 35ºC for 2 h. Next, deionized water (80 ml) was slowly dropped into the reaction system over a period of 30 min. Then, 200 ml of deionized water was added to dilute the mixture. In the final procedure, the mixture was treated with 30% H_2_O_2_ (20 ml) and the stirring continued for 10 min to obtain a GO suspension. During the centrifugation step, the dark brownish mixture was first repeatedly centrifuged and washed with deionized water until pH 6 was achieved.

To obtain small uniform GO sheets, low-speed centrifugation at 5000 rpm was first used to remove the thick multilayer flakes until all visible particles were removed (15 min). The supernatant was further centrifuged at 8000 rpm for 10 min to obtain the GO sheets. Each GO suspension (10 mg/mL in DI water) was subjected to ultrasonication using a probe type sonicator (Vibra-Cell VCX-500, 500 W, 20 kHz, Sonics & Materials, Inc., Newtown, CT, USA) at 25% amplitude for 15 min. An ice bath was used to avoid increasing the temperature during probe sonication.

Synthesis of PtNPs was carried out by reducing PtCl6^2-^ ions to PtNPs by mixing 10 mL of 1 mg/mL vanillin in 90 mL of 1 mM aqueous H_2_PtCl_6_.6H_2_O. The mixture was maintained at 100°C in a sealed flask to avoid evaporation during the 1 h on the hotplate, since the temperature determines the rate of the catalyzed reduction. For control experiments, the same amount of platinum solution and vanillin was maintained separately under the same reaction conditions. The reduced platinum solution was sonicated for 30 min to separate platinum nanomaterials from the biomolecules. After sonication, the solution was filtered using a 0.2 µm pore size syringe filter. The reduced platinum was purified by repeated centrifugation at 5,000 rpm for 30 min and the pellets were washed with distilled water to remove impurities.

Synthesis and characterization of the GO-PtNPs nanocomposite was done as described previously [20]. GO-PtNPs nanocomposites were prepared using vanillin as the reducing agent. To synthesize the nanocomposites, GO powder (1 mg/mL) was dispersed in ultrapure water (50 mL) by ultrasonication for 1 h. Then, 1 mM H_2_PtCl_6_.6H_2_O and 1 mg/mL vanillin was added concurrently to the brown GO solution and the mixture was vigorously stirred at 90 °C for 12 h after the color change from black to brown. The reaction was stopped and the mixture was washed three times with water using centrifugation. The pure product that was obtained was used for further experiments. All the samples were characterized as prescribed previously [8,19,21]. Ultraviolet-visible (UV-Vis) spectra of GO were recorded using an OPTIZEN POP spectrophotometer (Seoul, South Korea). X-ray diffraction (XRD) analyses were performed in a Bruker D8 DISCOVER X-ray diffractometer (Bruker AXS GmBH, Karlsruhe, Germany). The dried powders of GO were diluted with KBr to perform FTIR spectroscopy (Perkin Elmer Inc., USA), and spectrum GX spectrometry within the range of 500–4000 cm^−1^. The X-ray source was 3 kW with a Cu target; high-resolution XRD patterns were measured using a scintillation counter (*λ* = 1.5406 Å). The XRD was run at 40 kV and 40 mA, and samples were recorded at 2θ values between 10° and 80°. Raman spectra of GO were measured using a WITEC Alpha300 laser with a wavelength of 532 nm. Calibration was initially performed using an internal Si reference at 500 cm^-1^, yielding a peak position resolution of less than 1 cm^-1^. The spectra were measured from 500 to 4500 cm^−1^. A JSM-6700F semi-in-lens field emission scanning electron microscope (FESEM) was used to acquire scanning electron microscopy (SEM) images. The solid samples were transferred to a carbon tape held in an SEM sample holder, and the analyses were performed at an average working distance of 6 mm. Transmission electron microscopy (TEM; Hitachi H-7500, Seoul National University, South Korea) was used to determine the size and morphology of the GOs. TEM images of GOs were obtained at an accelerating voltage of 300 kV. All samples were deposited on glass slides in powdered form without a solvent.

2.3. Cell viability assay

LNCaP androgen-sensitive human prostate adenocarcinoma cells were cultured in RPMI 1640 medium (Gibco) supplemented with 10% (v/v) fetal bovine serum, 1% penicillin (v/v), and 1% streptomycin (v/v) (Sigma-Aldrich). The cells were grown to logarithmic growth phase and mixed with various concentrations of GO, GO-PtNPs, and PtNPs for 24 h, followed by determinations of cytotoxicity. Inhibitory concentration (IC50) is the concentration of PtNPs causing 50% inhibition of growth of the cells.

2.4. Cell proliferation assay

Cell proliferation was determined using bromodeoxyuridine/5-bromo-2'-deoxyuridine (BrdU) (Roche, Basel, Switzerland). The cells were incubated with various concentrations of GO, GO-PtNPs, and PtNPs for 24 h. BrdU labeling solution was added to the culture medium 2 h before the end of incubation. Cells were fixed and the level of incorporated BrdU was determined using the Cell Proliferation. Cell Proliferation was determined by incorporation of 5-bromo-2’-deoxyuridine (BrdU) into cellular DNA during cell proliferation using an anti-BrdU antibody. BrdU, this pyrimidine analog is incorporated in place of thymidine into the newly synthesized DNA of proliferating cells.

ELISA BrdU assay kit (Roche, Basel, Switzerland) following the manufacturer’s instructions. The proliferation activity of untreated cells at 0 h was considered as 100%.

2.5. Measurement of cytotoxicity

The membrane integrity of LNCaP cells was evaluated using an LDH Cytotoxicity Detection Kit (Sigma-Aldrich, St.Louis USA) according to the manufacturer’s instructions. Briefly, the cells were exposed to IC50 concentrations of GO, GO-PtNPs, and PtNPs for 24 h. Then, 100 μL of each cell-free supernatant was transferred in triplicate into wells of a 96-well plate, followed by 100 μL of the LDH reaction mixture. After 3 h of incubation under standard conditions, the optical density of the color generated was determined at a wavelength of 490 nm using a microplate reader.

2.6. Cell mortality assay

Cell mortality was evaluated using the trypan blue assay as described previously [7]. LNCaP cells were plated into wells of 6-well plates (1 × 10^5^ cells per well) and incubated for 24 h with IC50 concentrations of GO, GO-PtNPs, and PtNPs. Cells cultured in medium without GO, GO-PtNPs, and PtNPs were used as controls. Twenty-four hours later, the cells were detached with 300 µL trypsin–EDTA solution and both adherent and suspended cells were collected. The mixture of the supernatant and detached cells was centrifuged at 1200 rpm for 5 min. The pellet was mixed with 700 µL of trypan blue solution and dispersed. After 5 min of staining, the cells were counted using a cytometer. The viable cells were unstained and dead cells were stained blue. Three independent experiments were performed in triplicate. The mean and standard deviation were calculated. Cell proliferation is expressed as the percentage of viable cells relative to the appropriate control.

2.7. Determination of reactive oxygen species (ROS), malondialdehyde **(**MDA), nitric oxide (NO), and carbonylated protein levels

ROS were estimated as described previously [34]. Briefly, LNCaP cells were seeded into wells of 24-well plates at a density of 5 × 10^4^ cells per well and cultured for 24 h. After washing twice with phosphate-buffered saline (PBS), fresh medium containing IC50 concentrations of GO, GO-PtNPs, and PtNPs were added and incubated for 24 h. The cells were supplemented with 20 μM DCFH2-DA (Sigma—Aldrich, St Louis, USA) and incubation was continued for 30 min at 37°C. The cells were rinsed with PBS, 2 mL of PBS was added to each well, and the fluorescence intensity was determined using a Gemini EM spectrofluorometer (Molecular Devices, Sunnyvale, CA, USA) at an excitation wavelength of 485 nm and an emission wavelength of 530 nm.

MDA levels were determined using a thiobarbituric acid-reactive substances assay as previously described with suitable modifications [34]. LNCaP cells were seeded into six-well microplates at 2.0×10^6^ cells per well. The cells were treated with the respective IC50 concentrations of GO, GO-PtNPs and PtNPs for 24 hours. After incubation, the cells were harvested and washed twice with an ice-cold PBS solution. The cells were collected and disrupted by ultrasonication for 5 minutes on ice. The cell extract (100 μL) was used to detect MDA according to the procedure recommended by the manufacturer of the MDA assay kit (Sigma, St Louis, USA). The concentration of MDA was measured on a microplate reader at a wavelength of 530 nm. The protein concentration was determined using the Bio-Rad protein assay kit (Bio-Rad Laboratories Inc., Hercules, CA, USA).

NO production was quantified spectrophotometrically using Griess reagent (Sigma-Aldrich). The absorbance was measured at 540 nm and the nitrite concentration was determined using a calibration curve prepared with sodium nitrite as the standard [35].

Carbonylated protein content was measured according to Uehara and Rao [36]. Briefly, 10 μL of 1 μg/μL of protein lysate from each sample was denatured with 10 μL of 10% (w/v) sodium dodecyl sulfate (SDS) and derivatized with 20 μL of 20 mM 2,4-dinitrophenylhydrazine (DNPH) solution prepared in 10% (v/v) trifluoroacetic acid (TFA). After incubation at room temperature for 10 minutes with vortexing every 2 minutes, the reaction was neutralized with 20 μL of 2 M Tris base. A 3 μL aliquot of DNP-derivatized sample was diluted with 0.25 mL of adsorption buffer (20 mM NaHCO_3_, 150 mM NaCl, 0.25% SDS (w/v), pH 8.5), and 100 μL of diluted sample was loaded on to a 96-well plate. The sample wells were then incubated with 100 μL of blocking buffer containing goat anti-DNP antibody for 1 hour at room temperature. Following incubation, the sample wells were rinsed 6 times with PBST, and incubated with horseradish peroxidase (HRP)-conjugated rabbit anti-goat IgG antibody for 1 hour at room temperature. After washing 6 times with PBST, sample wells were incubated with 100 μL of TMB substrate at room temperature for 2–3 minutes for color development. The reaction was stopped with 100 μL of 0.5 M H_2_SO_4_ and the absorbance was measured at 450 nm and 690 nm. After subtraction of background absorbance at 690 nm, the carbonyl content in each sample was determined using a standard curve of oxidized BSA standard.

2.8. Mitochondrial membrane potential (MMP) determination

MMP was measured according to the manufacturer’s instructions (Molecular Probes, Eugene, OR, USA) using the cationic fluorescent indicator, JC-1 (Molecular Probes). LnCaP cells were incubated with 10 μM JC-1 at 37°C for 15 min, washed with PBS, resuspended in PBS, and then the fluorescence intensity was measured. MMP is expressed as the ratio of the fluorescence intensity of the JC1 aggregates to that of the monomers.

2.9. Measurement of ATP level

The ATP level was measured according to the manufacturer’s instructions (Catalog Number MAK135; Sigma-Aldrich) in LNCaP cells treated with IC50 concentrations of GO, GO-PtNPs, and PtNPs for 24 h. Decreased levels of ATP indicated cytotoxicity to the treated cells.

2.10. Measurement of anti-oxidative marker levels

The expression levels of oxidative and anti-oxidative stress markers were measured as described previously [37]. The anti-oxidative stress markers reduced glutathione (GSH), oxidized glutathione (GSH: GSSG) glutathione peroxidase (GPx), superoxide dismutase (SOD), catalase (CAT), and thioredoxin (TRX) were measured according to the manufacturer’s instructions (Sigma-Aldrich, St.Loius, USA). LNCaP cells were cultured in 75-cm^2^ culture flasks and exposed to IC50 concentrations of GO, GO-PtNPs, and PtNPs for 24 h. The cells were harvested in chilled PBS by scraping and washing twice with 1× PBS at 4°C for 6 min each at 1500 rpm. The cell pellet was sonicated at 15 W for 10 s (three cycles) to obtain the cell lysate. The resulting supernatant was stored at −70°C until analysis.

*2.11. Measurement of 8-oxo-dG and 8-oxo-G*

8-oxo-dG was determined as described previously [38] and also using the manufacturer’s instructions (Trevigen, Gaithersburg, MD, USA).

2.12 Reverse transcription-quantitative polymerase chain reaction (RT-qPCR)

Total RNA was extracted from the LNCaP cells treated with IC 50 concentrations of GO, GO-PtNPs, and PtNPs for 24 h using the PicoPure RNA isolation kit (Arcturus Bioscience, Mountain View, CA, USA). Samples were prepared according to the manufacturer’s instructions. Real-time RT-qPCR was conducted using a Vill7 device (Applied Biosystems, Foster City, CA, USA) and SYBR Green as the double-stranded DNA-specific fluorescent dye (Applied Biosystems). Target gene expression levels were normalized to the expression of *glyceraldehyde-3-phosphate dehydrogenase* (GAPDH) expression, which was unaffected by treatment. The real-time qRT-PCR primer sets are shown in Supplemental Table 1.

2.13. Statistical analysis

All assays were conducted in triplicate, and each experiment was repeated at least three times. The results are presented as the means ± standard deviation. All experimental data were compared using Student’s t-test. A p-value < 0.05 was considered statistically significant.
