# Supplementary material for: Graphene Oxide–Platinum Nanoparticle Nanocomposites: A Suitable Biocompatible Therapeutic Agent for Prostate Cancer
Source: Polymers (Basel). 2019 Apr 23;11(4):733. doi: 10.3390/polym11040733 (PMC6523086; doi:10.3390/polym11040733)
Supplement: Supplementary file 1 [file polymers-11-00733-s001.zip › Supplementary-467913/Table 1.docx]

| **Gene** | **List of primers** |
| --- | --- |
| **APEX1** | F:ATTGGCTGGAGGGCAGATCT |
|  | R:CCACTGGGTGAGGTTTTCTGA |
| **OGG1** | F:TCCTCCCTAGGTTTCCTCTC |
|  | R:TGAGACTAGTGACAGTGTTGG |
| **P53** | F:AGAGACCGTACAGAAGA |
|  | R:CTGTAGCATGGGATCCTTT |
| **P21** | F:GTTGCTGTCCGGACTACCG |
|  | R:AAAAACAATGCCACCACTCC |
| **Caspase-3** | F:AGGGGTCATTTATGGGACA |
|  | R:TACACGGGATCTGTTTCTTTG |
| **Caspase-9** | F:GTCACGGCTTTGATGGAGAT |
|  | R:CAGGCCTGGATGAAGAAGAG |
| **Bax** | F:CGAGCTGATCAGAACCATCA |
|  | R:GAAAAATGCCTTTCCCCTTC |
| **POLB** | F:GTTTCAGAAGAGGTGCAGAG |
|  | R:AGTGAAATAGAGAACACCACAG |
| **Bcl-2** | F:TAAGCTGTCACAGAGGGGCT |
|  | R:TGAAGAGTTCCTCCACCACC |
| **CREB1** | F:CAGTTCAGTCTTCCTGTAAGGACT |
|  | R:CGTTTGTCATGGTTAGTGTC |
| **UNG** | F:CTCTGCTTTAGTGTTCAAAGG |
|  | R:GAGTTCTGATTTAGCCAGGA |
|  | R:CCTTTGTACCGTTGCATCCT |
| **GAPDH** | F:AGGTCGGTGTGAACGGATTTG |
|  | R:TGTAGACCATGTAGTTGAGGTCA |

**Supplementary Table. 1**

| **CDK2** | **F:** GCTAGCAGACTTTGGACTAGCCAG |
| --- | --- |
|  | **R:** AGCTCGGTACCACAGGGTCA |
| **CDK4** | **F:** CTGGTGTTTGAGCATGTAGACC |
|  | **R:** AAACTGGCGCATCAGATCCTT |
| **GAPP45A** | **F:** TGCTCAGCAAAGCCCTGAGT |
|  | **R:** GCTTGGCCGCTTCGTACA |
