# Supplementary figures and images for: Graphene Oxide–Platinum Nanoparticle Nanocomposites: A Suitable Biocompatible Therapeutic Agent for Prostate Cancer
Source: Polymers (Basel). 2019 Apr 23;11(4):733. doi: 10.3390/polym11040733 (PMC6523086; doi:10.3390/polym11040733)

**Supplementary figure 1**

**
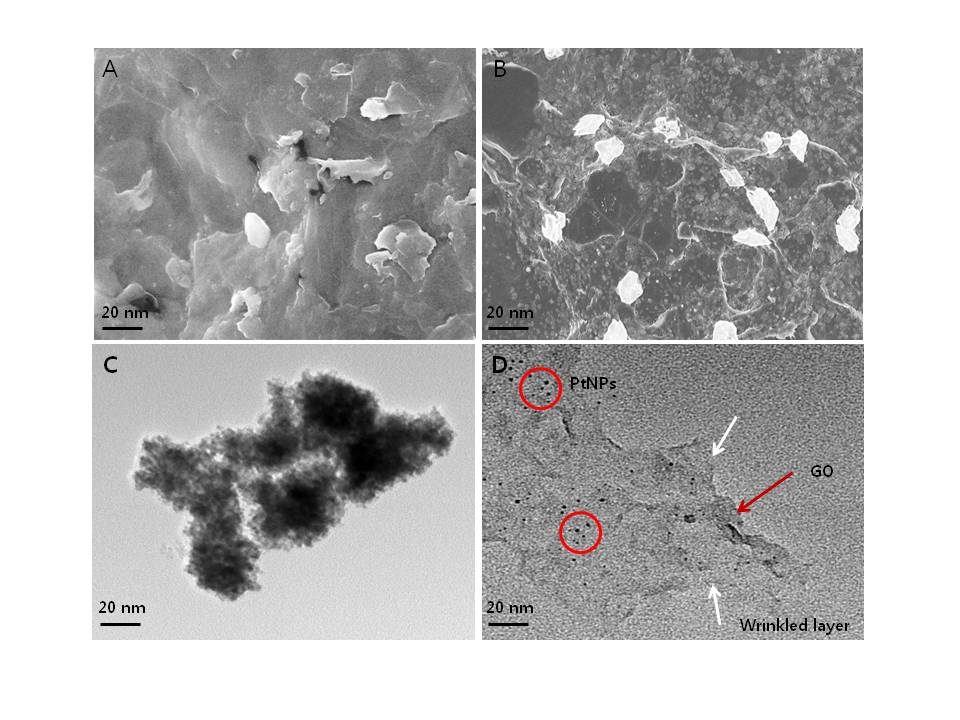
**

**
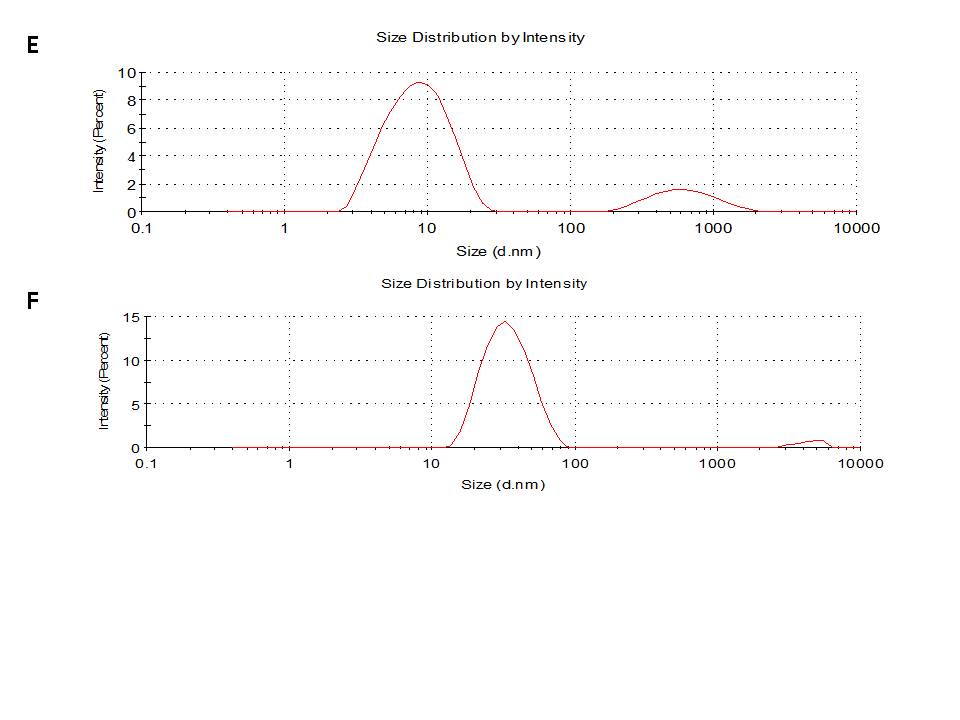
**

**Supplementary figure 2**

**
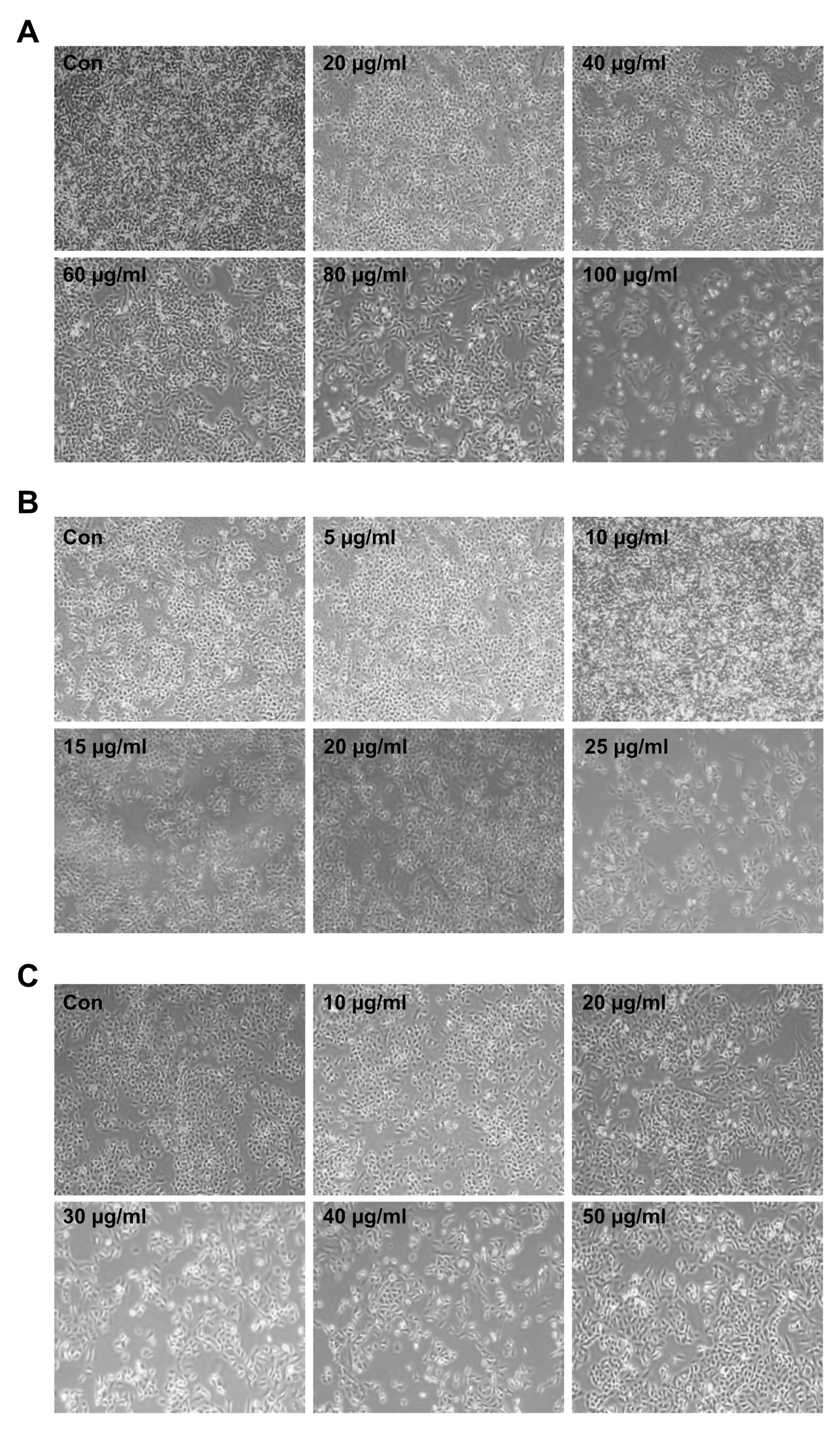
**

**Supplementary figure 3**

**
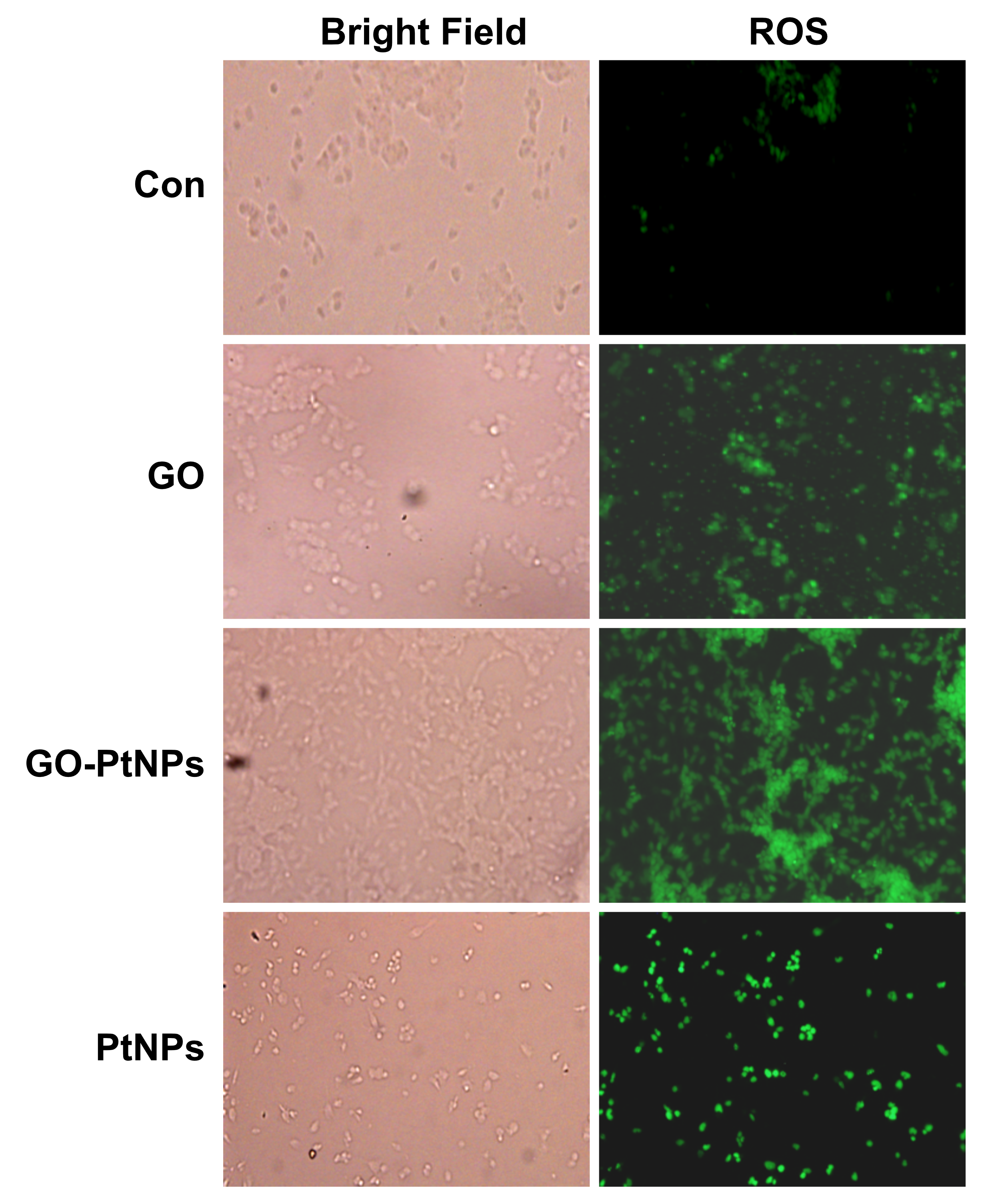
**

**Supplementary figure 4**

**
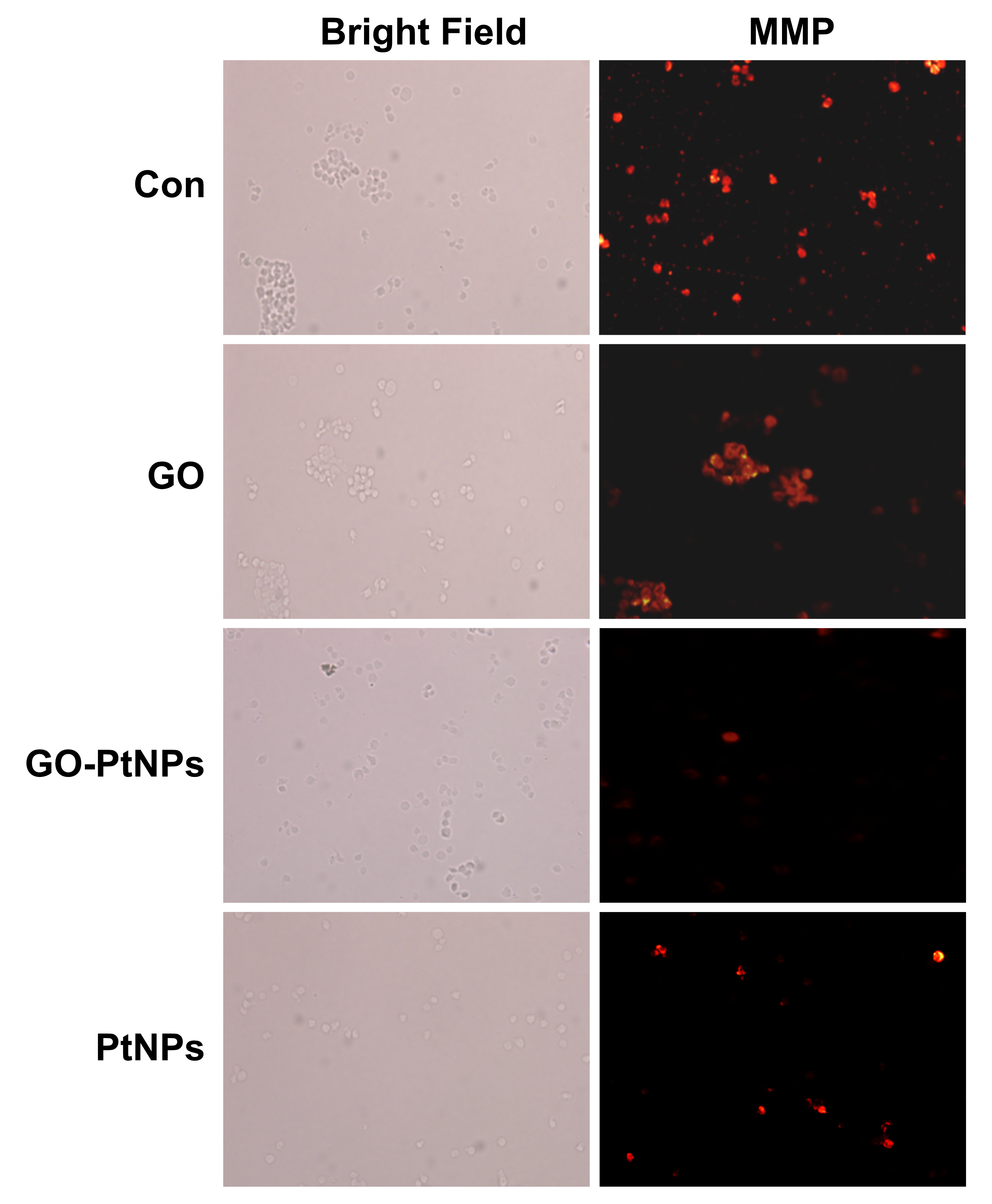
**

Supplement: Supplementary file 1 [file polymers-11-00733-s001.zip › Supplementary-467913/supplementary figure.docx]
